# Economic Impact of a Rotavirus Vaccine in Brazil

**DOI:** 10.3329/jhpn.v26i4.1880

**Published:** 2008-12

**Authors:** Dagna O. Constenla, Alexandre C. Linhares, Richard D. Rheingans, Lynn R. Antil, Eliseu A. Waldman, Luiz J. da Silva

**Affiliations:** 1 Department of Global Health, Rollins School of Public Health, Emory University, Atlanta, GA, USA; 2 Seção de Virologia, Instituto Evandro Chagas, Secretaria de Vigilância em Saúde, Ministério da Saúde, Av. Almirante Barroso 492, 66090-000 Belém, Pará, Brasil; 3 Departamento de Epidemiologia, Faculdade de Saúde Pública, Universidade de São Paulo, São Paulo, SP 01246-904, Brasil; 4 Disciplina de Infectologia, Departamento de Clínica Médica, Faculdade de Ciências Médicas, Universidade Estadual de Campinas, Campinas, SP, 13083-970, Brasil

**Keywords:** Costs and cost analysis, Diarrhoea, Infantile, Gastroenteritis, Morbidity, Rotavirus, Rotavirus vaccines, Vaccination, Brazil

## Abstract

The study was done to evaluate the cost-effectiveness of a national rotavirus vaccination programme in Brazilian children from the healthcare system perspective. A hypothetical annual birth-cohort was followed for a five-year period. Published and national administrative data were incorporated into a model to quantify the consequences of vaccination versus no vaccination. Main outcome measures included the reduction in disease burden, lives saved, and disability-adjusted life-years (DALYs) averted. A rotavirus vaccination programme in Brazil would prevent an estimated 1,804 deaths associated with gastroenteritis due to rotavirus, 91,127 hospitalizations, and 550,198 outpatient visits. Vaccination is likely to reduce 76% of the overall healthcare burden of rotavirus-associated gastroenteritis in Brazil. At a vaccine price of US$ 7-8 per dose, the cost-effectiveness ratio would be US$ 643 per DALY averted. Rotavirus vaccination can reduce the burden of gastroenteritis due to rotavirus at a reasonable cost-effectiveness ratio.

## INTRODUCTION

Rotavirus can lead to severe life-threatening gastroenteritis in infants and young children worldwide. Unlike many other enteropathogens, rotavirus affects children in both developed and developing countries (1-2) and is not associated with socioeconomic factors. However, the risk of dying from severe rotavirus-associated infection is higher in lower-income countries due to various factors, including limited access to healthcare facilities, malnutrition, earlier onset of infection, and mixed infections involving rotavirus and other pathogens. Rotavirus infects most children by three years of age, and prevention of rotavirus spread is virtually impossible, even in environments with the highest hygiene measures (2). Improvements in the availability of safe water and sanitation, access to oral rehydration therapy, and higher vaccine coverage have reduced the overall burden of gastroenteritis in children (3-4). From 1980 to 2002, Brazil showed a decrease by 91.5% in mortality due to gastroenteritis among children aged less than five years (3-4). During the same period, the proportion of infant deaths attributed to gastroenteritis fell from 41% to 4.7%, but with a range of 2.1% in the Southeast region to 7.8% in the Northeast (3-4). Infant mortality decreased from 85.2 per 1,000 children in 1980 to 25.1 per 1,000 children in 2002 (3-4).

Two surveys conducted in São Paulo in 1984-1985 and 1995-1996, including representative samples of the population aged 0-59 month(s), reported an overall reduction in the point prevalence of gastroenteritis from 1.7% to 0.9%, and a reduction in hospitalization due to gastroenteritis from 2.21 to 0.79 per 100 children-years (5). The latest survey (1995-1996) reported a 4.7% period prevalence of gastroenteritis when inquired about diarrhoea during the last two weeks (5). In contrast, a third survey carried out in the metropolitan area of Recife (Northeast region) in 1997 reported a 5.6% point prevalence of gastroenteritis and a period prevalence of 16.9% when inquired about diarrhoea during the last two weeks (6).

Despite improvements in health conditions among Brazilian children aged less than five years, gastroenteritis remains a significant burden in children in this age-group. In 2002, gastroenteritis caused about 3,000 deaths across the country, representing a rate of 19.6 deaths per 100,000 children aged less than five years, with a range of 34.2-7.6 deaths per 100,000 children in the Northeast and the Southeast respectively. Of the total deaths in children aged less than five years, infants were the most vulnerable group, with 81.8% of all deaths due to gastroenteritis (7).

Several studies have focused on the occurrence of acute diarrhoea associated with outpatient clinic visits and hospitalizations, yielding average prevalence rates that ranged from 12% to 42% throughout the country (8). An official nationwide surveillance system has been implemented through the Brazilian Health Ministry to determine the overall burden of rotavirus-associated disease and to monitor strain diversity. In March 2006, the Brazilian Health Ministry made available an attenuated vaccine against rotavirus-associated gastroenteritis for universal use in the country.

In this paper, we report the burden of disease and costs of gastroenteritis due to rotavirus in Brazilian children and the expected cost-effectiveness of a national rotavirus vaccination programme. The results presented here are based on a major study conducted in the region (9-10). Country-level estimates were used in the present analysis, and findings differ from the earlier study. In addition, this paper considers information on the recent introduction of the vaccine in the public sector in Brazil, including its cost.

## MATERIALS AND METHODS

### Model overview

A model was developed to estimate the disease outcomes and costs associated with gastroenteritis due to rotavirus in a hypothetical annual birth-cohort of children for a five-year period. The performance of the vaccination strategy is described using incremental cost-effectiveness ratios, defined as the additional cost of a specific strategy, divided by its additional benefit, compared with the next most costly strategy. Results are presented in US dollar (as in 2003) for local and regional decision-makers. Future costs and disability-adjusted life-year (DALY) estimates were discounted at an annual 3% rate as recommended by the US Panel of Cost-effectiveness in Health and Medicine (11) and the World Bank Global Burden of Disease Project (12). Model outcomes included averted hospitalizations, outpatient visits, deaths and DALYs, and costs. Costs, such as non-medical direct costs (transportation costs) or productivity losses to caregivers, were not included in the cost-effectiveness calculations; however, they were presented in the cost calculations.

The annual birth-cohort considered in the model included 3,471,000 children aged less than five years (13). The age distribution of rotavirus-associated disease was estimated for each of the disease outcomes using published findings of studies from Brazil and the Latin American region (14-23).

### Rotavirus-associated disease and economic burden

The burden of disease was estimated as the expected number of rotavirus-associated events (hospitalizations, outpatient visits, and deaths) during the first five years of life for a single birth-cohort. The risk of rotavirus-associated events were based on the cumulative risk of each event due to acute gastroenteritis during the first five years of life and the proportion of these events attributed to rotavirus. Detailed explanation of the methods used for estimating the burden of disease in Brazil can be found elsewhere (10).

In addition to estimating numbers of hospitalizations, outpatient visits and deaths, the burden of disease was also expressed in terms of DALYs (12). DALYs provide a standardized measure of the burden of disease that allows for cross-comparisons of the burden of disease and comparison with other diseases. The DALY estimate included years of life lost due to premature mortality and years lived with disability. The DALY loss from mortality was calculated based on the average country-specific life expectancy at zero and one year of age using life tables reported by the World Health Organization (WHO) (24). The average life expectancy for males and females is 68.2 at birth and 69.7 at one year of age. For the calculation of years lived with disability, only morbidity from disease severe enough to require medical care was considered. Default disability weights from the Global Burden of Disease Study (12), the WHO guidelines for cost-effectiveness studies (25), and estimated rotavirus illness duration of six days (26) were used in calculating years lived with disability. DALYs calculated reflect the disabili-ty weight and duration estimates for gastroenteritis due to rotavirus as provided in the Global Burden of Disease Study (12). This calculation is age-weighted (25) and uses an annual discount rate of 3%.

The economic burden of rotavirus-associated gastroenteritis for Brazil was estimated by combining estimates of the number of each type of event with information on the costs associated with the event. Country-specific estimates of healthcare costs were developed for hospital and outpatient rotavirus-associated events. In addition to healthcare costs, transportation costs to healthcare facilities were also included in the calculations of costs. For the base case analysis, these costs were based on assumptions made about the number of trips to and from the doctor's office and/or the hospital and the cost of each trip. The number of trips to and from the doctor's office and/or hospital was based on the amount of outpatient visits and hospital admissions reported by physicians in a multicentre hospital-based rotavirus surveillance study (Abate H, Linhares AC, Venegas G, Vergara R, Lopez, P, Jimenez E *et al*. Results of a hospital-based study of rotavirus gastroenteritis in Latin American children. Resumen presentado en el Congreso Internacional de Pediatría en Cancún, México, 15-20 de agosto de 2004. Unpublished) conducted in Belem. These estimates were considered to be the most reliable estimates for Brazil. Indirect costs associated with lost time from paid work were also calculated. For the base case analysis, these costs were based on the number of days off from work (one outpatient consultation or one day in the hospital was assumed to be the same as one day off from work), assuming an average 2003 minimum monthly salary in Brazil and 22 days off from work. A complete description of the methods used in calculating the economic burden was reported elsewhere (10).

### Effectiveness of vaccination and costs

Estimates of the effectiveness of rotavirus vaccination were based on the results of clinical trials of human rotavirus vaccine administered orally to infants at two and four months of age (27-28). The vaccine demonstrated an 85% efficacy in preventing hospitalizations for gastroenteritis due to rotavirus (27). Efficacy in averting deaths was assumed to be the same as that for hospitalized cases (85%). No data on specific efficacy are available for outpatient visits. Therefore, rates for the episodes of severe gastroenteritis due to rotavirus were considered (84.7-86%) to be rotavirus-associated gastroenteritis (27-28). For the baseline analysis, the efficacy of one dose was assumed to be 62.5% [95% confidence interval [CI] (16-83) (López P, Linhares AC, Pérez-Schael I, Ruiz-Palacios GM, Costa-Clemens SA, Sanchez N *et al*. Early protection against severe rotavirus gastroenteritis—RIX4414 experience in Latin America. European Society of Paed Infect Dis Congress, May 3-5 2003, Basel, Switzerland. Unpublished). It was further assumed that efficacy would not decline in the second year (Linhares. Personal communication, 2007) and subsequent years following vaccination.

We assumed that children would receive the vaccine to prevent rotavirus-associated gastroenteritis at the time of the diphtheria-tetanus-pertussis-hepatitis B virus-*Haemophilus influenzae* vaccine (DTPwHBV/Hib) and the oral polio virus vaccine (OPV) in Brazil, which are given at two, four, and six months of age. In the baseline analysis, the national coverage of the third dose of DTPw at one year of age for 2003 was estimated to be 96% (29). Rotavirus vaccination would occur with DTPwHBV/Hib and OPV doses (one and two); however, standardized data are only available for the coverage of the third dose.

In this analysis, we included the cost of administration of the vaccine, the price of each dose, the number of doses given (based on coverage level), and expected losses from waste (10%). The costs for administering the vaccine included the cost of healthcare personnel and training, cold-chain, storage space, and public education. Brazil has the necessary infrastructure for running a national rotavirus vaccination programme, based on its extensive history of administering oral polio vaccine. Therefore, the incremental administration costs are assumed to be low. A few studies estimated the cost of immunization for current EPI vaccines (30-33); however, no data on the incremental cost of adding a vaccine to the current EPI regimen were found. Based on the range of estimates found in studies conducted on the immunization cost and the assumption of low incremental costs, the model assumes the cost for administering the vaccine as US$ 0.50 per dose. The Brazilian Ministry of Health purchased the vaccine at a cost of US$ 7-8 per dose and made it available for the public sector in March 2006.

Table 1 summarizes the best estimates used in the analysis.

**Table 1.** Input variables for a cost-effectiveness analysis of a rotavirus vaccination programme in Brazil

Variable	Baseline estimate	Source of data
Demographics		
Birth-cohort	3,471,000	PAHO, 2004 (13)
Life expectancy at birth (years)		
Male	68. 2	WHOSIS, 2000 (24)
Female	69.7	
Cumulative risk of events for rotavirus-associated gastroenteritis per 1,000 (<5 years)		
Hospitalization rate	35	
Ambulatory visit rate	199.5	Rheingans *et al.,* 2006 (10)
Mortality rate	0.7	Rheingans *et al.,* 2006 (10)
Direct medical cost of rotavirus-associated gastroenteritis per patient (US$)*		
Hospitalized cases	150.97	Rheingans *et al.,* 2006 (10)
Ambulatory cases	10.81	
Non-medical direct costs (transport) (US$)		
Hospitalized cases	1.49	Rheingans *et al.,* 2006 (10)
Ambulatory cases	0.04	
Indirect costs		
Hospitalized cases	18.58	Rheingans *et al.,* 2006 (10)
Ambulatory cases	8.35	
Vaccine		
Coverage	96%	WHO-UNICEF best estimates, 2003 (29)
Efficacy (outpatient visits due to rotavirus)	85%	Ruiz-Palacios *et al.,* 2006 (27) Salinas *et al.,* 2005 (28)
Efficacy (hospitalizations due to rotavirus)	85%	Ruiz-Palacios *et al.,* 2006 (27)
Efficacy (death due to rotavirus)	85%	Assumes efficacy is same as for hospitalizations (27)
Administration cost (US$ per dose)	0.50	Author's assumption
Vaccine price (US$ per dose)	7-8	Brazilian Ministry of Health, 2006 (purchase price)

*These costs include the direct medical costs of hospitalization, outpatient visits, diagnostic tests, and medication; PAHO=Pan-American Health Organization; UNICEF=United Nations Children's Fund; WHOSIS=World Health Organization Statistical Information System

### Sensitivity analyses

One-way sensitivity analyses were done by calculating the main outcomes, the economic burden, and cost-effectiveness, for different scenarios that are likely to influence the costs of rotavirus-associated disease and the cost-effectiveness of a vaccination programme. These scenarios included: high and low end-estimates of outpatient visits, hospitalization and mortality rates, vaccine efficacy against hospitalizations and death, hospital per diem, cost of outpatient visit, and price of vaccine.

## RESULTS

### Burden of disease

Table 2 shows the projected disease outcomes of rotavirus in Brazil under current treatment (no vaccination) and with rotavirus vaccination. By the age of five years, one in five (205 per 1,000) children required a clinic visit for rotavirus-associated gastroenteritis, one in 29 (35 per 1,000) children was hospitalized for gastroenteritis due to rotavirus, and one in 1,429 (0.7 per 1,000) children died due to rotavirus-associated gastroenteritis. An estimated 24 DALYs per 1,000 births were lost from these outcomes. This compares with the rates observed in other countries in the region where rates of rotavirus-associated mortality are low (34-35). For the base case, the model predicts that a total of 550,198 outpatient-visits (159 per 1,000), 91,127 hospitalizations (26 per 1,000), and 1,804 deaths (0.52 per 1,000) associated with gastroenteritis due to rotavirus would be prevented by rotavirus vaccination. This represents an overall reduction of 77%, 76%, and 73% respectively.

**Table 2.** Estimated burden of rotavirus in Brazil with and without vaccination programme

Type of event	Total events	Events per 1,000 children
Current management (without vaccination)		
Hospitalizations	120,513	34.72
Outpatient visits	712,249	205.20
Deaths	2,475	0.71
DALYs*	83,365	24.02
With vaccination		
Hospitalizations	29,386	8.47
Outpatient visits	162,051	46.69
Deaths	671	0.19
DALYs*	22,671	6.53
Benefit of vaccination (averted events)†		
Hospitalizations	91,127	26.25
Outpatient visits	550,198	158.51
Deaths	1,804	0.52
DALYs*	60,694	17.49

*Discounted at 3%;

†Benefit of vaccination is based on the vaccine effectiveness, which incorporates information on the vaccine efficacy and coverage; DALYs=Disability-adjusted life-years

### Costs of events due to rotavirus-associated gastroenteritis

Estimates of the direct medical costs, direct non-medical costs, and indirect costs for inpatients and outpatients with gastroenteritis are provided in Table 3 and are based on the hospital-based surveillance. The total direct medical cost for inpatients was US$ 150.97, with 86% of the cost attributed to the hospital stay and the remainder due to the cost of diagnostics and medication. For outpatients, the total direct medical cost was only US$ 10.81, of which 50% is attributed to the cost of the visit.

**Table 3.** Costs of treating gastroenteritis in Brazil estimated from hospital-based surveillance data*

Variable	Inpatients (n=43)†	Outpatients (n=52)†
	Mean	Range	Mean	Range
Cost per stay/visit (US$)	129.76	35.07-280.56	5.39	
Medications (US$)	14.47	1.64-62.25	4.87	0.22-33.98
Diagnostics (US$)	6.74‡	0-14.97	0.55	0-12.50
Total direct medical costs (US$)	150.97‡	36.71-357.78	10.81	0.22-46.48
Percentage who paid to visit child	44		6	
Payment to visit child (US$)	1.49‡	0-8.77	0.04‡	0-1.30
Mean number of trips	8.58	0-48	0.5	0-6
Non-medical direct costs (US$) (transport)	12.78‡	0-1,161	0.02‡	0-31.50
Percentage who lost time from work	53		48	
Hours lost from work	28.90¶	5-108	19.92¶	2-120
Average female hourly wage (US$)	1.45§		1.45§	
Indirect costs (US$)	41.90		28.88	
Total cost per patient (US$)	205.65		39.71	

*10 sites participated in the health economic component of the hospital-based surveillance study: 2 secondary-level hospitals, 5 primary healthcare units (Unidades Basicas de Saude [postos de saude]), 2 private healthcare units (emergency rooms linked to tertiary-level hospitals), and one Unidad del Programa de Familia Saludable;

†Cost data are based on US$ as in 2003;

‡Missing one value;

¶Median value calculated for those parents who indicated they lost time from work;

§This value assumes an eight-hour shift

Forty-four percent of caregivers reported that they paid to visit the child at the hospital. For all caregivers (including those who did not report payment per visit), the mean cost to transport child to the hospital was US$ 1.49. This was multiplied by the mean number of trips (8.58) for a total of US$ 12.78 per child. For outpatients, 6% of caregivers reported that they paid to visit the child at the outpatient clinic. Of those who paid, the median cost to transport the child was US$ 0.04. This was multiplied by the mean number of trips (0.5) for a total of US$ 0.02 per child.

The indirect cost associated with lost wages for inpatients was more than that for outpatients (US$ 41.9 vs US$ 28.9) because the percentage of subjects who lost time from work was higher for caregivers of inpatients than outpatients (53% vs 48%) and, on average, caregivers of inpatients lost more time from work than outpatients (28.9 hours vs 19.9 hours).

Table 4 shows the estimated healthcare costs per birth-cohort from these rotavirus-associated events in Brazil. In the absence of vaccination, it is estimated that rotavirus-associated gastroenteritis results in a total healthcare cost of over US$ 25.3 million for each annual birth-cohort in Brazil, which is equivalent to US$ 7.30 per child. Seventy percent of these costs are associated with hospitalization. Vaccination is likely to reduce the economic burden of gastroenteritis due to rotavirus significantly in Brazil, averting US$ 19.3 million (76% of the total healthcare cost).

**Table 4.** Estimated healthcare costs* (US$) and benefits of a rotavirus vaccination programme in Brazil

Type of event	Direct medical cost*	Mean cost per child*,†
Current management (without vaccination)		
Hospitalizations	17,813,038	5.13
Outpatient visits	7,519,460	2.17
Total	25,332,498	7.30
With vaccination		
Hospitalizations	4,350,341	1.25
Outpatient visits	1,713,689	0.49
Total	6,064,030	1.75
Benefit of vaccination (averted costs)‡		
Hospitalizations	13,462,697	
Outpatient visits	5,805,771	
Total	19,268,469	

*Values are based on US$ as in 2003;

†Calculation only includes direct medical costs to the healthcare system;

‡Benefit of vaccination is based on the vaccine effectiveness, which incorporates information on the vaccine efficacy and coverage

### Cost-effectiveness of a rotavirus vaccination programme

The expected costs and benefits of a rotavirus vaccination programme in Brazilian children are presented in Table 5. Results shown here are for a single birth-cohort, assuming 96% coverage and a basic vaccine price of US$ 7-8 per dose. Costs of the vaccination include the cost of the vaccine and its administration. Moreover, vaccination cost assumes only the cost of vaccinating children who would receive the vaccine, based on vaccine coverage estimates and 10% wastage. Benefits are expressed as net medical costs from the healthcare system perspective and the cost-effectiveness ratio. All of these cost and benefit measures vary directly with the vaccine price.

**Table 5.** Estimates of costs, net benefits, and cost-effectiveness of a rotavirus vaccination programme in Brazil

Vaccine programme cost*	Amount† (US$)
Vaccine administration	3,332,160
Vaccine (US$ 7-8/dose)	54,980,640
Total cost	58,312,800
Net medical direct cost	39,044,331
Incremental cost-effectiveness ratio	
Cost per DALY	643
Cost per life saved	21,643
Cost per hospitalization averted	428
Cost per medical visit averted	70

*Vaccination cost assumes only the cost of vaccinating children who would receive the vaccine, based on vaccine coverage estimates and 10% wastage;

†The intervention cost, net medical cost, and incremental cost-effectiveness ratio (US$/DALY) assume a vaccine price of US$ 7-8 per dose. Values are based on US$ as in 2003; DALY=Disability-adjusted life-year

At a price of US$ 7-8 per dose, the net medical cost for the healthcare system to vaccinate the Brazilian birth-cohort with the rotavirus vaccine would be US$ 39 million. From the healthcare perspective, a rotavirus vaccination costs US$ 643 per DALY averted, US$ 21,643 per life saved, US$ 428 per hospitalization averted, and US$ 70 per medical visit averted. The vaccination programme would be considered cost-saving to the healthcare system at a vaccine price lower than US$ 2.17 per dose. Higher vaccine prices require a net economical investment but provide a health benefit.

### Sensitivity analysis

The estimated medical cost per child is most sensitive to changes in assumptions regarding the incidence of hospitalization due to rotavirus and the cost of hospital treatment. A 25% change in either of those parameters results in a 12-15% change in the medical cost per child. The estimates of incremental cost-effectiveness rate are affected by the rotavirus-associated mortality rate, efficacy of vaccine against mortality, and vaccine price. A 25% change in any of these variables results in 15-40% change in the incremental cost-effectiveness ratio. Overall, an increase in the incidence of rotavirus and efficacy of vaccine will result in a lower incremental cost-effectiveness ratio making a vaccination programme more cost-effective. Furthermore, a reduction in the price of rotavirus vaccination (US$ 5 per dose) will yield a lower incremental cost-effectiveness ratio of US$ 341 per DALY averted, making the vaccine even more cost-effective.

## DISCUSSION

This is the first time a national rotavirus vaccination programme is evaluated in Brazil in economic terms. Rotavirus-associated gastroenteritis is a common disease with an estimated 832,762 cases of gastroenteritis due to rotavirus and 2,475 deaths occurring annually in Brazil. The large burden of outpatient visits due to rotavirus-associated gastroenteritis is a significant contributor to the substantial costs in the healthcare system.

Vaccination provides an effective opportunity for improving children's health in Brazil. We estimated that vaccination would prevent more than three-fourths of all cases due to rotavirus-associated gastroenteritis, including 1,804 deaths. This translates into almost one life saved and 185 cases prevented per 1,000. We also estimated that vaccination would result in total medical savings of US$ 19.3 million. At the current vaccine price of US$ 7-8 per dose, vaccination would be cost-effective from the perspective of healthcare system based on the WHO benchmarks for cost-effectiveness.

It is important to emphasize that even if vaccination appears to be cost-effective according to a criterion of the cost-effectiveness ratio being less than the per-capita GDP of Brazil, it may not be affordable. Even an intervention that provides good value for resources invested may have prohibitive financial requirements that could not be accommodated by the healthcare system of Brazil.

Limitations of this analysis include, but are not limited to, uncertainties around the epidemiology of gastroenteritis due to rotavirus in Brazil and efficacy of vaccine against death. Moreover, the actual proportion of diarrhoeal deaths due to rotavirus is unknown. Efforts should be made to ascertain the incidence of deaths due to rotavirus-associated gastroenteritis using active surveillance studies, especially in regions of the country where there may be limited access to emergency treatment facilities.

Another limitation is related to the coverage rate and/or timing of vaccination. The coverage is based on the coverage of other vaccines, e.g. DTPwHBV/Hib and OPV, which may not be an accurate estimation of coverage for the current rotavirus vaccine. In the analysis, we assumed that all groups within the country have equal likelihood of vaccination, and all children would receive the vaccine at the recommended time. If high-risk populations were missed or vaccination was delayed, the effectiveness would be reduced. Since rotavirus-associated gastroenteritis occurs in young children and protection is conferred from the time of vaccine dose 1 until dose 2 is given (29), it is important that the vaccine be given on time; this is the scenario adopted in the health economic model used for this analysis. Future considerations should be made to account for the fact that not all children will receive the vaccine at the recommended time.

A final limitation is the lack of data on the magnitude of herd immunity which may be conferred by the partial coverage of vaccination into a population. The analysis considered the direct effects of vaccination, but it did not consider the indirect protective effect on persons never vaccinated. The herd immunity effect could be large and might offset inefficiencies in the delivery of a complete course and vaccination on time to all children. This analysis supports the conclusion that gastroenteritis due to rotavirus poses a sizeable burden in Brazil and results in 120,513 hospitalizations, 712,249 outpatient visits, 2,475 deaths, and 83,365 DALYs annually in Brazil. At a price per dose of US$ 7-8 for Brazil, the cost per DALY averted is less than US$ 700. This ratio is less than per-capita GDP of Brazil, but more importantly, it compares favourably with the ratios of other vaccines (36). For example, the cost-effectiveness of pneumococcal conjugate vaccination ranged from US$ 110 to US$ 2,150 across a range of countries (37-38); rotavirus vaccination ranged from US$ 290 to US$ 12,300 in Latin American countries (9,34-35,39-40); and influenza vaccination was cost-saving in high-risk children in Argentina (41).

## ACKNOWLEDGEMENTS

Funding for the study was provided by GlaxoSmithKline Biologicals. Full independence of methods and control over publication remain with the authors along with responsibility for any errors. The authors thank the following experts for their assistance in identifying epidemiological data and their general support for the study: Alfredo Gilio (Diretor da Divisaõ de Pediatria, Clínica of Hospital Universitário, Saõ Paulo); Expedito Luna (Diretor, Departamento de Vigilância Epidemiológica, Brasilia); Divina das Dôres de Paula Cardoso and Paulo da Costa (Instituto de Patologia Tropical e Saúde Pública, Universidade Federal de Goiás, Goiânia, Goiás); Marcos Bosi Ferraz (Centro Paulista de Economía da Saúde, Saõ Paulo); and Nilo Serpa (Analista de Sistemas, Secretaria de Saúde do Estado de Rio de Janeiro).
